# Psychiatric Features of Parents of Children with Spina Bifida

**DOI:** 10.1192/j.eurpsy.2023.1231

**Published:** 2023-07-19

**Authors:** V. Ozer, P. Ulual, I. Alataş, R. Çetiner, D. Uğurlar, F. Izci, O. Güçlü

**Affiliations:** 1 Istanbul Basaksehir Cam ve Sakura Sehir Hastanesi; 2 İstanbul Bilim Üniversitesi; 3 İstanbul Eğitim ve Araştırma Hastanesi, İstanbul, Türkiye

## Abstract

**Introduction:**

Spina Bifida (SB) is a closure defect of the neural tube. Affecting multiple systems of the body, this disease also affects families psychologically.

**Objectives:**

In this study, our aim was to investigate levels of psychiatric symptoms, depression, anxiety, despair and coping with stress in parents of children with Spina Bifida.

**Methods:**

From the follow-up patients’ families of our hospital’s neurosurgery unit, a total number of 80 parents were included in this study. Sociodemographic data form, The Structured Clinical Interview -Clinical Version (SCID-I / CV) for DSM-IV Axis Diagnosis, Beck Anxiety Inventory (BAI), Beck Depression Inventory (BDI), Symptom Checklist (SCL-90-R), Beck Hopelessness Scale (BHS) and Coping with Stress Scale were performed.

**Results:**

The mean age of parents of children with Spina Bifida diagnosis was 34.44±7.00. Psychiatric symptoms and inventory scores are displayed on the table.

**Table 1:** Clinic Inventory Scores of Cases

	Mean	Standard Deviation
Scl-90	0,86	0,63
Beck Depression Inventory	13,00	10,32
Beck Anxiety Inventory	12,93	11,71
Beck Hopelessness Scale	5,30	3,74
Coping with Stress	52,94	8,53

**Conclusions:**

It was determined that psychiatric symptoms such as anxiety, depression, difficulty in coping with stress can be seen among parents of children with SB. This suggests that parents of patients with diseases like SB should get the needed psychiatric help and supportive care during the course of treatment.

**Disclosure of Interest:**

None Declared

